# Clinical Features and Epidemiological Insights of Acute Epidemic Conjunctivitis: A Multicentric Cross-Sectional Study in North Central India

**DOI:** 10.22336/rjo.2024.68

**Published:** 2024

**Authors:** Ankita Aishwarya, Amit Agarwal, Deepti Saxena, Vaibhav Jain, Adarsh Singh, Rachna Agarwal

**Affiliations:** 1 Department of Ophthalmology, Sanjay Gandhi Post Graduate Institute of Medical Sciences, Lucknow; 2 Agarwal Netralaya, Aishbagh, D.A.V College Market, Lucknow; 3 CBMR, Sanjay Gandhi Post Graduate Institute of Medical Sciences, Lucknow

**Keywords:** epidemic conjunctivitis, pink eye, eye infection, viral conjunctivitis, haemorrhagic conjunctivitis, eye flu, AEC = Acute Epidemic Conjunctivitis, PAN = Pre-auricular Lymph node involvement, EKC = Epidemic Keratoconjunctivitis, PCF = Pharyngoconjunctival Fever, AHC = Acute Hemorrhagic Conjunctivitis, IRB = Institutional Review Board

## Abstract

**Purpose:**

To gain epidemiological insights by investigating the age, risk factors, and clinical features of individuals affected by the conjunctivitis outbreak.

**Methods:**

The study was conducted at various ophthalmic centers, involving participants with clinical symptoms of acute conjunctivitis within one week from 15 June 2024 to 15 July 2024. Demographic information, clinical features, signs, and symptoms were recorded and analyzed using SPSS version 21.0 and MedCalc software.

**Results:**

The study included 920 patients (1722 eyes), 56% males and 44% females, and most cases were bilateral (94%). Among the affected groups, hospital staff (43%) were the most affected, followed by school-going children (31%), those in direct contact with infected individuals (17%), or others (11%). The median age of onset was 26 years, with a range spanning from 2 to 76 years. The age group most affected was 19-49 years (52%), followed by <18 years (34%) and the elderly group (14%). The most common and first symptom was foreign body sensation (92%), and additional symptoms included ocular itching (81%) and watering (80%). The most prevalent signs were conjunctival congestion (99%), follicles (96%), subconjunctival bleeding (43%), eyelid swelling (51%), chemosis (39%), and pre-auricular lymph node enlargement (6%). Corneal involvement was not observed.

**Discussions:**

Acute Epidemic Conjunctivitis (AEC) outbreaks have significant implications for public health, particularly regarding healthcare resource utilization, economic burden, and disruption of daily life. Timely identification, effective communication, and coordinated response strategies are essential to managing AEC outbreaks and preventing their escalation.

**Conclusion:**

This study provides crucial insights into epidemic conjunctivitis in North Central India. The study findings can guide targeted interventions and healthcare resource allocation to manage the outbreak effectively.

## Introduction

Acute Epidemic Conjunctivitis (AEC), commonly known as “pink eye,” is an infectious and highly contagious condition affecting the conjunctiva. The most common cause of AEC is viral infections, accounting for 80% of the cases [1]. Major outbreaks can manifest as epidemic keratoconjunctivitis (EKC), pharyngoconjunctival fever (PCF), and acute hemorrhagic conjunctivitis (AHC) [2-7]. This ocular infection is highly contagious and poses a significant public health concern due to its rapid transmission, leading to the potential for widespread outbreaks, particularly in crowded areas and close-knit communities [8].

The epidemiological findings show that EKC and AHC were prevalent in Asia during the early winter and spring. At the same time, PCF circulated mainly in China, Australia, and the United States during the summer [8-11]. In a study conducted in 2020, Das et al. found that elevated levels of rainfall, wind speed, and humidity were associated with a reduced incidence of EKC cases throughout the year [12].

In the last month, several regions around the country have experienced outbreaks, leading to increased attention from the medical community and public health authorities. Understanding the burden and clinical presentation in a population is crucial for better care and preparedness during outbreaks and to prevent the significant economic burden associated with the loss of school days and its resulting ocular morbidity.

## Methods

This cross-sectional observational study was conducted at three different healthcare facilities in Uttar Pradesh: a referral tertiary care center at Raebareilley Road, primary health care at Kanpur-Lucknow Road, and a private center near the railway station. The study included all patients presenting with clinical symptoms of acute conjunctivitis within one week from 15 June 2023 to 15 July 2024. Patients with a history of ocular surface disorder, corneal ulcer, or trauma were excluded from the study.

Patients or their parents/guardians provided informed consent for participation and picture disclosure. Detailed demographic data, symptomatology, duration of symptoms, medical history, and potential risk factors were recorded using a structured questionnaire. Ophthalmologists performed comprehensive eye examinations, including assessment of visual acuity, lymph node involvement, eyelid swelling, conjunctiva, or corneal involvement. The study adhered to the Declaration of Helsinki. Our study was granted an exemption from full IRB review by the Institutional ethics committee Sanjay Gandhi Postgraduate Institute of Medical Science (Number: 2024-153-IP-EXP-60), as it falls under the category of minimal risk research and involves a depersonalized case study. The institutional ethics committee approved the research design.

### Sample size

The study included all the patients who fulfilled the inclusion criteria, a total of 920 patients.

Data were analyzed using descriptive statistics to summarize the characteristics of the study population. Independent t-tests, One-way ANOVA, and Chi-square tests were used to assess associations between variables, with a p-value<0.05 considered statistically significant. Data analysis was done using SPSS 23.0 and MedCalc software.

## Results

The study included 1722 eyes of 920 patients. Four hundred forty-two patients were reported from tertiary centers, 352 from primary health centers, and 126 from the private sector. Overall, there were 518 (56%) males and 402 (44%), and 862 (94%) cases were bilateral. The mean duration of symptoms ranged from 48 ± 120 hours. The demographics of three different centers are presented in **Table 1**. The median age of onset was 26 years, with a range spanning from 2 to 72 years. Notably, the Private Centre had a higher proportion of younger participants (≤ 18 years) and a significant urban population. The distribution of occupations and exposure categories also varied among the centers, with hospital staff being the predominant group in the Tertiary Health Centre and school children and individuals in direct contact being prominent in the other centers. These demographic differences provided essential insights into the distribution of acute conjunctivitis cases across different segments of the population and healthcare settings.

**Table 1:** Demographic characteristics of study participants across different health centers

	Tertiary Health Centre (N=442) x (%)	Primary Health Centre (N=352) y (%)	Private Centre (N=126) z (%)
≤ 18years	126 (28.5)	112 (31.8)	74 (58.7)
19-49 years	245 (55.4)	189 (53.7)	47 (37.3)
≥ 50 years	71 (16.1)	51 (14.5)	05 (4.0)

Male (N=518)	234 (45.2)	196 (37.8)	88 (17.0)
Female (N=402)	208 (51.2)	156 (38.8)	38 (10)

Urban	242 (54.8)	129 (36.6)	123 (97.6)
Rural	200 (45.2)	223 (63.4)	03 (2.4)

Hospital staffs (N=398)	213 (53.5)	162 (40.7)	23 (5.8)
School Children (N=285)	116 (40.7)	84 (29.5)	85 (29.8)
Direct contact (N=156)	54 (34.6)	86 (55.1)	16 (10.3)
Others* (N=81)	59 (72.8)	20 (24.7)	02 (2.5)

* Swimming pool users and other gathering areas like religious places and restaurants. The numbers within each cell represent the counts of participants, while the percentage within parenthesis represents the proportion of participants within each category. Hospital staff (43%) represented the most significantly affected group, while the age range most affected was 19-49 years (52%).

**Fig. 1:**
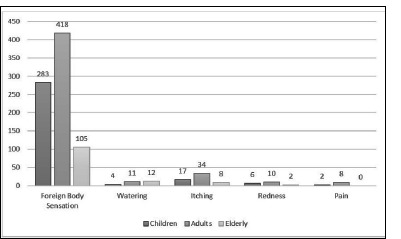
Illustration of the primary presenting symptoms among various age groups, with the most prevalent initial complaint being a foreign body sensation overall

**Fig. 2:**
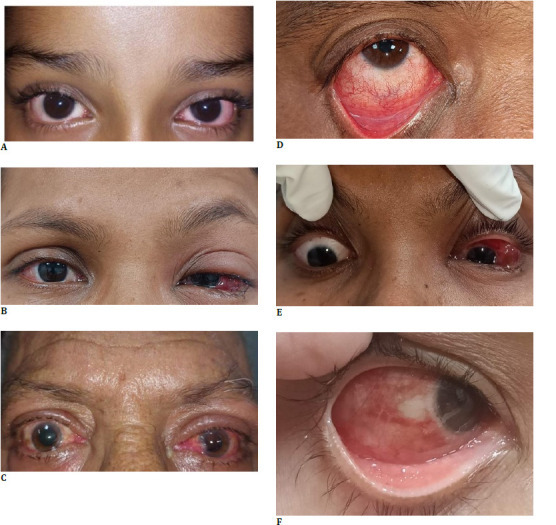
Clinical features among different age groups, including bilateral conjunctival hyperemia in a 6-year-old child (**A**), bilateral conjunctival hyperemia with left eye edema in a 39-year-old woman (**B**), bilateral conjunctival hyperemia with mild left eye upper lid edema in a 67-year-old man (**C**), inferior conjunctival congestion with pseudomembrane and follicular reaction (**D**), left eye chemosis (**E**), and diffuse subconjunctival hemorrhage (**F**)

**Table 2** comprehensively analyzes reported symptoms among different age groups, namely children, adults, and older people. Notable variations in symptom prevalence were observed across the groups. An Independent t-test revealed significant findings for Foreign body sensation and redness of the eye (p<0.0001), watering, and itching (p<0.012). Foreign body sensation and pink eye were more prevalent in all age groups; however, fever and discharge were rare. Pain showed a consistent prevalence, with around 65% reporting it in all three groups. The itching had a relatively lower prevalence in older people (56%) compared to children (72%) and adults (73%). Fever was rarely reported, with the highest prevalence among children (0.9%). **Fig. 1** highlights variations in primary symptom reporting across different age groups, with foreign body sensation being the first symptom in all age categories. Watering, itching, redness, and pain showed varying degrees of prevalence among the different age groups. **Table 3** comprehensively compares clinical features concerning acute conjunctivitis within distinct age groups (children, adults, and elderly). Notably, conjunctival hyperemia and follicular reaction were widespread across all age categories, exhibiting statistical significance. Results from an Independent t-test confirmed a significant association between conjunctival hyperemia and follicular reaction (p<0.0001), subconjunctival hemorrhage (p=0.0003), and eyelid swelling (p=0.0483). Conversely, the prevalence of subconjunctival hemorrhage, chemosis, and preauricular lymph node enlargement exhibited no substantial variation, while eyelid swelling highlighted prominence among children. **Fig. 2** illustrates various clinical presentations with parts labeled as a, b, c, d, e, and f, respectively. Subconjunctival hemorrhage was more predominantly observed in the age group between 19 to 49 years. Eyelid swelling was more prominent in the < 18- or > 50 age group.

**Table 2:** Prevalence of eye symptoms across different age groups

	Children n=312 (%)	Adults n=481 (%)	Elderly n=127 (%)	p-value
Foreign body sensation	298 (96)	454 (94)	99 (78)	**< 0.0001**
Redness	286 (92)	440 (91)	97 (76)	**< 0.0001**
Watering	208 (67)	395 (82)	91 (72)	**0.012**
Pain	196 (63)	311 (65)	82 (65)	0.75
Discharge	103 (33)	121 (25)	30 (24)	0.297
Itching	225 (72)	351 (73)	71 (56)	**0.0012**
Fever	3 (0.9)	1 (0.2)	0	0.864

Foreign body sensations, itching, and redness were more common in children and adults than in older people. Pain, discharge, and fever did not exhibit significant disturbances among the age groups.

**Table 3:** Comparative analysis of ocular signs among different age groups

	Children n=312 (%)	Adults n=481 (%)	Elderly n=127 (%)	p-value
Conjunctival Hyperemia	312 (100)	481 (100)	124 (97.6)	**< 0.0001**
Subconjunctival Haemorrhage	83 (27)	301 (62)	57 (44.9)	**0.0003**
Eyelid Swelling	208 (66.7)	185 (38.5)	72 (56.7)	**0.0483**
Chemosis	98 (31.4)	205 (42.7)	59 (46.5)	0.148
Pseudomembrane	56 (18)	99 (20.5)	21 (16.5)	0.24
Keratitis	0	0	0	
PAN*	32 (10.3)	03 (0.6)	21 (16.5)	0.41
Follicles	299 (95.8)	479 (99.6)	106 (83.5)	**< 0.0001**

* PAN = Pre-auricular Lymph node involvement While clinical features demonstrated overall similarity across all three age groups, children and older people exhibited a higher prevalence of eyelid swelling and enlarged pre-auricular lymph nodes. None of the participants had corneal involvement.

The overall reported p-values underscored notable disparities in clinical features across the various age groups, accentuating the significance of age-related analysis in comprehending the diverse manifestations of conjunctivitis. Moreover, the initial complaint of foreign body sensation should serve as a crucial indicator, raising heightened awareness regarding the potential likelihood of acute epidemic conjunctivitis.

## Discussion

Acute epidemic conjunctivitis is a highly contagious eye condition often occurring in outbreaks or epidemics. Adenovirus accounts for 15% to 70% of total cases of infectious conjunctivitis, followed by coxsackie virus [13-15]. Frequently misdiagnosed as bacterial conjunctivitis, these cases often result in improper use of antibiotics, leading to worsening antimicrobial resistance [16]. In recent years, several epidemics have been reported in various regions, prompting concern in the medical community due to their potential to spread and rapidly affect a significant portion of the population. The highly contagious nature of these infections is underscored by the viability of Human Adenovirus 19 on various surfaces, remaining viable for up to 8 days on paper, 9 days on tonometer tips, 10 days on textiles and metal, and up to 35 days on plastics [17]. This extended viability contributes to its infectiousness on surfaces for 4-5 weeks, ultimately amplifying the likelihood of frequent nosocomial transmission [18].

Seasonal changes can also lead to a higher prevalence of allergic conjunctivitis, which shares some symptoms with acute epidemic conjunctivitis, such as redness and itching. Allergic conjunctivitis exhibits a seasonal pattern, typically surging in April and tapering by June, as shown in China, the USA, and West Africa [19-21]. The co-occurrence of allergic conjunctivitis during certain seasons might complicate the diagnosis of AEC, potentially leading to delays in appropriate management.

Statistical modeling suggests a peak occurrence of adenoviral conjunctivitis cases typically observed from July to September, echoing trends in the USA, Japan, and Germany [11,22-24]. Our study similarly identified a July peak during the epidemic. However, this pattern diverges from research conducted in Turkey [25], where a higher prevalence was noted from April to May. Additionally, Das et al.’s study [12] in Hyderabad revealed a peak epidemic keratoconjunctivitis prevalence in April, contrasting with its lowest prevalence in July. This variation may arise from distinct viral strains and geographic and climatic disparities [26,27]. The elevated prevalence of allergic conjunctivitis during this period might contribute to its masking as EKC. Additionally, Das et al. [12] employed an electronic medical record system for data collection; although explicit details regarding inclusion or exclusion criteria were not specified, neither were clinical features considered. Similarly, the study in Turkey [25] lacked essential epidemiological information such as gender, age, and geographical data. The higher incidence of acute epidemic conjunctivitis (AEC) observed in July could be influenced by factors related to seasonal changes, environmental conditions, and human behavior. The exact reasons may vary depending on the specific region and circumstances or some potential explanations: rainfall often leads to higher humidity levels, which can prolong the survival of infectious agents on surfaces and in the environment. This can facilitate the transmission of conjunctivitis through contaminated hands, objects, or surfaces. During rainy periods, people may seek shelter indoors, increasing crowding in confined spaces. This proximity can promote the transmission of conjunctivitis. Rainy weather can hinder the effectiveness of specific public health measures, such as maintaining dry and clean environments. This could affect the containment of conjunctivitis outbreaks, especially in places with inadequate sanitation or where preventive strategies are more complex.

These outbreaks are frequently linked to densely populated environments, including schools, workplaces, and communities where close contact facilitates the transmission of the causative agents [1,10,28,29], mirroring the findings of our study. The inherently contagious nature of acute conjunctivitis underscores the imperative for robust public health measures to curb its propagation and mitigate its repercussions.

The age group most affected in our study was 19-49 years (52%), followed by < 18 years (34%), and the elderly group comprising 14% with a median age of onset at 26 years (range 2 to 76 years). This contrasted with the findings of Liu et al. in China [29], which indicated that the onset age of acute hemorrhagic conjunctivitis was predominantly concentrated in individuals aged 0-20 years and 60 years. In contrast, the median age remained comparable, which aligns with Zhang’s study, which reported a median onset age of 24 years [30].

This divergence could be attributed to variations in selection criteria and participant numbers, given that the study specifically focused on cases of acute hemorrhagic conjunctivitis. Nevertheless, the overall range remained comparable to the study by Zhang et al. [9], in which vulnerable age groups between 4 and 85 years were susceptible to EKC infections during the outbreaks.

Global occurrences of conjunctivitis outbreaks attributed to adenoviruses have been documented across various parts of the world [9,31]. EKC and AHC are prominent forms of conjunctivitis, particularly among Asians, while PCF outbreaks have been reported in tropical and subtropical areas of Australia, the USA, and China [9]. The first symptom seen in most patients in our study, irrespective of age group, was foreign body sensation. This study marks the first reporting of the earliest symptom among acute epidemic conjunctivitis patients. This observation might be attributed to the obstruction of blood circulation to the conjunctiva [29], aiding in recognizing an alerting sign to consider acute conjunctivitis before commencing therapy. Common clinical manifestations encompassed conjunctival congestion, follicular reaction, subconjunctival hemorrhage, eyelid swelling, and chemosis, exhibiting similar findings in diverse studies [29,32,33]. Corneal involvement in EKC is joint, presenting in various forms, including punctate keratitis, subepithelial infiltrates, and reduced corneal sensation [34-38]. None of our patients had corneal involvement possibility due to the selected cases being within one week of symptom onset and variations in viral strains, as these studies solely focused on epidemic keratoconjunctivitis.

AEC outbreaks have significant implications for public health, particularly regarding healthcare resource utilization, economic burden, and disruption of daily life. Timely identification, effective communication, and coordinated response strategies are essential to managing AEC outbreaks and preventing their escalation. Preventing the spread of epidemic conjunctivitis is crucial to containing outbreaks. Implementing strict hygiene practices, such as frequent handwashing and avoiding direct contact with infected individuals, can help reduce transmission [39]. Health professionals should wear protective eyewear and consider taking leave if infected as a precaution [40]. In settings where AEC outbreaks are occurring, temporary closure of affected institutions and education about personal hygiene can contribute to controlling the epidemic. Surveillance systems that monitor the prevalence and patterns of AEC can aid in early detection and intervention, thus minimizing the impact on affected communities. The effects of seasonality on epidemic conjunctivitis are multifaceted, with a combination of climatic, behavioral, and environmental factors influencing the occurrence and spread of AEC outbreaks. Ensuring enough chlorination levels is imperative to prevent epidemics of conjunctivitis linked to the swimming pool environment.

This analysis had several limitations of a cross-sectional study. Although these data may not be generalizable to the entire country and could be subject to misclassification errors, this study lacked molecular data such as viral genotype.

## Conclusion

To summarize, acute epidemic conjunctivitis is a contagious eye condition that can lead to outbreaks with potential public health implications. Minor symptoms like foreign body sensations should not be ignored as these might be the earliest indicators of conjunctivitis. Understanding the epidemiology, clinical presentation, diagnostic challenges, treatment options, and preventive measures is vital for healthcare professionals and policymakers to effectively manage and mitigate the impact of AEC epidemics. Further research and collaboration are needed to develop comprehensive strategies for early detection, containment, and prevention of AEC outbreaks.
